# A Study on the Effects of Cognitive Overloading and Distractions on Human Movement During Robot-Assisted Dressing

**DOI:** 10.3389/frobt.2022.815871

**Published:** 2022-05-03

**Authors:** Antonella Camilleri, Sanja Dogramadzi, Praminda Caleb-Solly

**Affiliations:** ^1^ Bristol Robotics Laboratory, University of The West of England, Bristol, United Kingdom; ^2^ Department of Automatic Control and Systems Engineering, University of Sheffield, Sheffield, United Kingdom; ^3^ School of Computer Science, University of Nottingham, Nottingham, United Kingdom

**Keywords:** human movement, safety, human robot interaction, close proximity, assistive dressing, cognition, collaborative behaviour

## Abstract

For robots that can provide physical assistance, maintaining synchronicity of the robot and human movement is a precursor for interaction safety. Existing research on collaborative HRI does not consider how synchronicity can be affected if humans are subjected to cognitive overloading and distractions during close physical interaction. Cognitive neuroscience has shown that unexpected events during interactions not only affect action cognition but also human motor control Gentsch et al. (Cognition, 2016, 146, 81–89). If the robot is to safely adapt its trajectory to distracted human motion, quantitative changes in the human movement should be evaluated. The main contribution of this study is the analysis and quantification of disrupted human movement during a physical collaborative task that involves robot-assisted dressing. Quantifying disrupted movement is the first step in maintaining the synchronicity of the human-robot interaction. The human movement data collected from a series of experiments where participants are subjected to cognitive loading and distractions during the human-robot interaction, are projected in a 2-D latent space that efficiently represents the high-dimensionality and non-linearity of the data. The quantitative data analysis is supported by a qualitative study of user experience, using the NASA Task Load Index to measure perceived workload, and the PeRDITA questionnaire to represent the human psychological state during these interactions. In addition, we present an experimental methodology to collect interaction data in this type of human-robot collaboration that provides realism, experimental rigour and high fidelity of the human-robot interaction in the scenarios.

## Introduction

Physical human-robot interactions are complex and require synchronized human-robot movements. Synchronicity requires intention recognition and prediction of the human movement. Research in cognitive neuroscience has shown that disruption in human movements can occur with external disturbances, not only affecting action cognition but also motor control Gentsch et al. (2016). In order for the robot to safely adjust and adapt its trajectory in response to distracted human behaviors, prior knowledge of quantitative changes in the human movement can be essential. If the behavior of the human is disrupted, then this disruption needs to be investigated further to ensure safe and timely adaptive interactions. Such prior knowledge would benefit from ensuring that any movement adaptation is implemented in a safe context and not in an instance in which the collaborative state of the human is disrupted. Understanding how a change in behavior (due to cognitive overloading and distractions) affect human movement in the context of robot-assisted dressing is an important research problem that could lend insight for improving physical HRI and addressing safety concerns. It is difficult to predict how the movement will be disrupted, but we can monitor deviations from the expected trajectory and the loss of the human-robot synchronicity. While there is other research in this area investigating similar interaction contexts, such as Pignat and Calinon (2017), Erickson et al. (2019) and Koganti et al. (2017), these do not consider the effect of unexpected events on human behavior, and adaptation of the robot movements assumes consistent human behavior at all times.

Robot-assisted dressing in our study involves the bi-manual Baxter robot helping a person put on a jacket. Tracking human arms just before physical contact with the garment ensures a correct starting position for dressing. When the hand is in the sleeve, the robot trajectory can be guided by, e.g. force feedback as described by Chance et al. (2016). However, before the hand enters the sleeve, it can be hard to achieve physical coupling between the opening of the sleeve and the human hand. At this initial stage of the dressing task, when humans can move their arms freely in a shared workspace, the coupling relies only on trajectories executed by the robot and human. At this point, disruptions in human movement, which could be due to external disturbances, need to be modeled so that the robot can adapt appropriately. Even though it is possible to do adaption through feedback control when there is no direct physical contact Erickson et al. (2019), identifying these non-collaborative instances has a priority transcending the execution of the reference dressing trajectory. Related works about the psychology of humans performing joint tasks show the importance of synchronicity to achieve cooperation Sebanz et al. (2003), Sebanz et al. (2006), Ciardo and Wykowska (2018). Such literature state that poor synchronization (non-collaborative instances) between movements is perceived as an uncooperative partner, and as a result, it affects the representation of the shared task. Therefore, it is crucial to identify how human motor control is affected in the instance where external disturbances are to occur during an assistive task.

To study the effect of the disruption, we designed a controlled experiment to obtain a reliable set of data and model the impact of various external disturbances. These disturbances were created in the form of unexpected events, carefully timed to disrupt the human movement before the hand makes contact with the jacket and deviates from the expected trajectory. From recording the human and robot movements, we evaluated differences between the expected and disrupted human trajectory. This allowed us to analyze and quantify the disrupted human movement, producing data that we used to model lack of collaboration. The NASA Task Load Index (TLX) was used to measure perceived workload and the PeRDITA to represent participants’ perceived experience. These two sets of qualitative user experience results support the quantitative data of human movements. This research is the first step towards prediction and adaptation in close physical human-robot interaction during realistic situations where the human could be distracted. This paper provides the following contributions: 1) Design of an experimental HRI methodology which includes timed interruptions to expose changes in the collaborative interaction during a robot-assistive dressing task; 2) An analysis of the changes comprising qualitative evaluation of the user experience showing how *cognitive overloading* and *distractions* increased the cognitive workload; and 2) The quantitative analysis of the effect of the change in collaborative behavior on human movement when exposed to unexpected events during a robot-assisted dressing task.

The paper is organized as follows: In Section 2, we review related literature regarding human movement, adaptation in the context of physically assistive Human-Robot Interactions (HRI) and the relationship between action cognition and motor control. Section 3, describes the experimental procedure and methodology used to collect data during the controlled HRI experiment. In Section 4, we present qualitative and quantitative results obtained from the HRI experiment. Finally, in Section 5, we discuss our findings, main contributions and recommendations for future work.

## 2 Related Works

### 2.1 Human Movement in Close-Proximity Collaborative Tasks

Synchronized human and robot motion is essential for the success of collaborative tasks, but it can be disrupted by external factors. Humans have capabilities and limitations that can either complement or hinder the completion of physically collaborative tasks. The work of Haddadin et al. (2011) suggests that the robot’s collaborative mode can only be deemed safe if the human’s collaborative intention is taken into account during the task. Potential disruptions of the collaborative human state need further investigation to ensure safe and graceful disruption recovery and, facilitate adaptive robot behavior. The existing literature reviewed assumes that human behavior either correctly adapts to robot movements or remains consistent during interactive tasks. Our concern is that collaborative human movement can lead to disruptions and loss of synchronicity in certain situations. Literature on close-proximity interactions focuses on task completion through continuous adaptation to human movements without considering its potential discontinuity, which can be expected in different dynamic environments.

Collaborative human-robot interactions are typically addressed through prediction and adaptation by the robot, whereas the human collaborative state is assumed constant Ben Amor et al. (2014), Ewerton et al. (2015), Pignat and Calinon (2017), Vogt et al. (2017). Prediction in HRI relies on evaluating the current interaction state and choosing correct actions Hoffman (2010), Schydlo et al. (2018). In Hoffman (2010) a reactive and anticipatory action selection is compared. The anticipatory approach combines the current state with a probabilistic view of the temporal activity, providing better efficiency over the reactive approach Hoffman (2010). The work presented by Ben Amor et al. (2014) involves interaction primitives that combine the probabilistic temporal view of the movement variation with performed adaptation. Both Hoffman (2010) and Ben Amor et al. (2014) state that the robot is interacting with an engaged human. Therefore, in these works, the correct anticipatory action can only be selected with high confidence if there is mutual responsiveness and commitment to the collaborative task; if not, the human’s non-collaborative state can pose a safety risk.

In other related studies, which are focused on physical contact, Erickson et al. (2019), adaptation of the robot movements considers changes in human movement, but changes in human behavior (distinct to specific movements) due to distractions or cognitive loading are not addressed. In an assistive dressing task, human movement is in close proximity with the robot, and for safety, it requires high confidence in predicting and adapting the robot’s movement. Distractions and failure are very likely in a real-life context, and therefore needs to be a clear understanding of changes to human engagement and movement. Robot-assisted dressing failures have been considered by Chance et al. (2016), but analysis and modeling of the human’s collaborative state in the presence of disruptions were not included. In this paper, we visualize and model discontinuity of the human’s collaborative state through human movement observations. Through a series of controlled HRI experiments, we observed variations, limitations, and differences in human movements in the presence of disruptions during a collaborative task. Disruptions included in the experiment were based on relevant literature on human behavior, action cognition and motor control. In a robot-assisted dressing context, physical interactions start as soon as human limbs are inside the dressing garment. When humans are exposed to distractions and cognitive overloading, their collaborative state can change even before the physical interaction starts and impact their movements. The change to the synchronicity cannot be modeled using a probabilistic approach or be recognized as yet another movement primitive, as shown in Dermy et al. (2017), especially if the probabilistic models are not trained on a disrupted human movement data set as shown in Wang et al. (2013). Similarly, modeling of human motion uncertainty has only been performed for collaborative tasks. The works of Yamazaki et al. (2014), Gao et al. (2015), Yu et al. (2017), Joshi et al. (2019) and Koganti et al. (2019) model this uncertainty in close-proximity collaborative tasks in specific scenarios in which either a global trajectory is learned or motor skills are encoded. However, the human movement modeled in these studies are for general skills to perform a task without any considerations of disrupted human movement.

The non-linearity and high dimensionality features comprising human behavior can be challenging to investigate. For human movement analysis, dimensionality reduction is used to express the limb-based characterization in a more readable space. This methodology takes advantage of visualization to spot disruption within the human movement as a change in the collaborative behavior. Latent variable models (Orrite-Urunuela et al., 2004) are used to address these challenges (Zhang et al., 2017; Joshi et al., 2019) to model limitations in human movement or to personalize human movements. This paper, will use a related approach to model the human movement and highlight any disruptions associated with the change in collaborative behavior. The method used is the Gaussian Process Latent Variable Model (GP-LVM), which is a bayesian non-parametric model which acts as a dimensionality reduction method by using a Gaussian Process (GP) to learn a low-dimensional representation of high-dimensional data. The advantage of using such a method is using the non-linear learning characteristic of GP, which is ideal for human movement Zhang et al. (2017). The non-parametric model properties allow a distribution-free form model with a flexible structure that can scale to accommodate the complexity of the dataset.

### 2.2 Human Behavior, Action Cognition and Motor Control

To instigate a lack of collaboration and observe its effect on the interaction, understanding human cognition and mental model is necessary. In this paper, we hypothesize that a change in a human’s collaborative state can lead to a change in human movement. This hypothesis is based on neuroscience research defined as action cognition that amalgamates human motor control, perception, and cognition Gentsch et al. (2016) and can be mathematically formulated to describe human adaptive behavior as a resistance to a natural tendency to disorder. One principle is the free-energy principle which states that the human brain actively makes observations while concurrently minimizing the world’s model Friston (2010). When an unexpected event occurs, the equilibrium obtained from the minimized free-energy model will be disrupted. The human reaction would be to minimize the differences between their free energy world model and the world updates brought by their senses and associated perception. The work presented in Friston (2010) suggests that human movement gets disrupted in such a case to minimize the differences in the interaction. Disruptions during task performance, and hence the world model, can be due to *cognitive overloading* and/or environment *distractions* as defined by Orru and Longo (2019). Cognitive load is the amount of information that a person can hold in their working memory at a given time Friston (2010), Gentsch et al. (2016), Orru and Longo (2019). Memory can be classified into short-term, long-term, working and sensory memory. Sensory memory perceives and preserves auditory and visual cues in the short term memory. On the other hand, working memory takes new information and organizes it among already learned information that is stored in the long-term memory Friston (2010), Gentsch et al. (2016), Orru and Longo (2019). Long-term memory is effectively limitless, unlike the working memory, which is essential for learning and performing a task. When unexpected events occur during a physically assistive task, the working memory has to process new information, which consequently increases the cognitive load.

**FIGURE 1:**
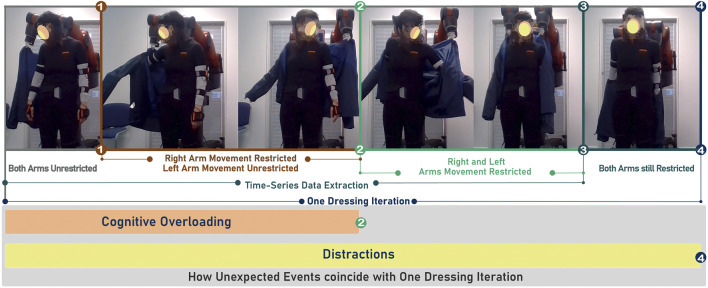
One dressing task timeline. Markers from (1) to (4) highlight the different stages during the assistive task. The first image on the far LHS shows the moment in the dressing task when the right arm is unrestricted. The following two images show the dressing with the left arm unrestricted. The two images between Markers (2) and (3) show the instance where the left hand becomes restricted, followed by an image where both hands become restricted. After Markers (3), the robot drags the jacket back down the arms of the participant. The orange block represents the time during which *cognitive overloading* occurred. The yellow block represents the time during which *distractions* occurred. For all three parts of the experiment the dressing task was repeated ten times.

We hypothesize that cognitively overloading humans in human-robot collaborative tasks will result in disrupted synchronicity of their physical interaction. We consider how *cognitive overloading*, as well as *distractions* will unbalance the overall cognitive load made up of intrinsic, extraneous and germane loads. As such we also consider how the timing of unexpected events should be staged to trigger increased mental effort. Our HRI experiments explore this hypothesis, and Figure 1 shows their temporal layout. The distractions (staged unexpected events) were based on the mental models and loads further explained in Section 3.

## 3 Methodology

### 3.1 Experiment Setup and Procedure

**FIGURE 2:**
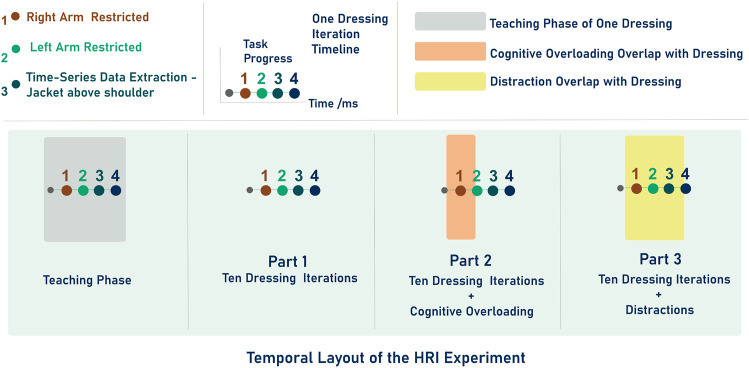
Overall experiment timeline after marker one: 1) teaching phase followed by Part 1 made of ten dressing tasks, 2) Part 2 consisting of ten dressing tasks with *cognitive overloading* and 3) Part 3 again consisting of ten dressing tasks with *distractions*. Markers from one to four represent the same instances in the dressing task as shown in Figure 1.

Our controlled HRI experiment was set up to demonstrate and study disruptions to collaboration during a robot-assisted dressing task in the presence of cognitive overloading and distractions (unexpected events). Our experimental procedure is shown in Figure 1 and Figure 2. We used a bi-manual Baxter research robot to perform pre-recorded dressing trajectories while a jacket was held by the gripper. The jacket was moved from the participant’s hand to their elbow. Subsequently, the jacket was pulled towards the participant’s left-hand side to allow them to insert their left hand/arm in the left sleeve. All dressing iterations are shown in Figure 1. One iteration of the dressing task is completed when the jacket reaches the participants’ shoulders. The robot then pulls the jacket down and prepares for the next dressing iteration. The whole process is shown in Figure 1 and will be referred to as one dressing iteration in this paper. The researcher chose when to start the first dressing iteration.

The entire experiment is divided into three parts with an initial learning sequence of one dressing iteration to familiarize participants with the task. The overall temporal layout of the experiment is shown in Figure 2. In the Parts Two and Three, we introduced unexpected events to disrupt human collaborative state. In Part Two the disruption to the collaborative task is the *cognitive overloading*, whereas in, Part Three the disruption is in the form of environment *distractions*. A monitor was placed in front of the participants to display letters that participants had to memorize as part of the *cognitive overloading*. The letters appeared at four different positions (from the LHS to the RHS) on the monitor, and participants had to memorize four consecutive letters as they appear (and then disappear) from the monitor. When the fourth letter was displayed, they had to say the four letters in their order of appearance. The letters that appeared on the monitor were always different. In Part Three of the experiment, there were two types of distractions, one was created by sounding a fire alarm, and the other was random questioning of the participants. In total, each participant had two distractions that occurred during one dressing iteration, as shown in Figure 2. The color-coded markers, numbered one to four, in Figure 1 represent different sequences of one dressing task and are also shown in the overall temporal layout in Figure 2. Marker one represents an initial position of the jacket at the right-hand side of the participant when participants were inserting the right arm in the jacket sleeve. Marker two represents the robot positioning the jacket close to the participant’s left hand and inserting the left arm until it becomes constrained in the jacket. The *cognitive overloading* was applied until marker two, as highlighted in Figure 1 because the participant’s arms were still not entirely restricted by the jacket at that stage. Marker three shows when the robot end-effector reached both shoulders. At marker four, the robot starts to pull the jacket down and out of the participant’s hands.

As noted earlier in Section 2, our working memory is used to recover already learned knowledge stored in the long-term memory. In Part One, participants used relevant knowledge on how to collaborate in the assistive dressing task acquired in the initial learning stage. In Part Two, new information related to the unexpected events had to be processed, which increased their cognitive load and led to disruption in the collaborative state (Orru and Longo, 2019). Part Two and Three further unbalanced this cognitive load and disrupted the efficient storage of the new information (Orru and Longo, 2019). Participants had to continuously modify their collaborative task plan based on the initially acquired knowledge of the task. To efficiently process new information in our working memory, we have to balance the cognitive load. For effective learning, the intrinsic cognitive load must be managed, extraneous cognitive load minimized, and germane cognitive load maximized. These three loads make the overall cognitive load. Intrinsic cognitive load is related to new information that needs to be processed to complete a task. The extraneous cognitive load Orru and Longo (2019) involves searching for information while trying to learn a task. The cost of processing information goes against the process of learning. Whereas, the germane cognitive load is described as an effort to construct a mental model of the task.

The Intrinsic cognitive load is often managed by good instructional sequencing, and in our controlled experiment, it is prompted by instructing participants to carry out an additional task during the collaborative dressing task. In Part Two, the letters appearing on the monitor were continuously changing with no obvious pattern. This increased intrinsic cognitive load due to a lack of proper instructional sequencing since it required a higher mental effort to process new information. The *distractions* in Part Three that included a fire alarm further increased intrinsic cognitive load. It was hypothesized that *cognitive overloading* and *distractions* would lead to inefficiency in performing the task since the intrinsic load was not managed. In our experiment, extraneous loading is triggered by asking participants to remember and say the four letters in the order of appearance, marked as *cognitive overloading*. In Part Three, this was implemented by posing questions to the participants and triggering a new information process. These distractions introduced new tasks that prevented using the initially acquired knowledge of the collaborative task. Therefore, the extraneous load was not minimized at these instances, requiring a higher mental effort from the participants. The temporal layout of the experiment was constructed to manipulate the germane load. The unexpected events do not allow participants to use an already built mental model of the task from Part One therefore, the maximization of the germane load got disrupted. This overall experimental structure allowed us to analyze the change in the human’s collaborative state through quantitative data collection of the human movement.

### 3.2 Human Movement Data Collection

Participants performed the dressing task in ten iterations in each part of the experiment as shown in Figure 1. An experiment information sheet was provided before the start, explaining the dressing task and the *cognitive overloading* of part two. The *distractions* used in Part Three were not included in the information sheet. In total, 18 participants took part in the experiment, aged 18 to 24 (4 participants) and aged 25 to 34 (14 participants). All of them had completed higher education. The experiments generated a data set of 540 dressing tasks. The dataset includes the right and left robot end-effector poses, forces and torques, and participants’ pose features. The data recorded from the robot and participants resulted in a time-series data set with a dimension size of 753,910 by 206 features.

We recorded human movement using a motion capture XSens suit Roetenberg et al. (2007) to obtain 23 joint positions and orientations on the participant’s body. The XSens suit provides a set of inertial measurement units that, together with bio-mechanical models and sensor fusion algorithms, can instantly validate data output. The joints recorded were the pelvis, spine, sternum, neck, head, collar bones, shoulders, elbows, hands, hips, knees, heels and toes, creating a data set of 161 features (7 readings per joint) at the frequency of 50 Hz. Participants were asked to take part in the calibration of the motion capture suit before the start of each part. For the calibration process the height, shoulder width, arm lengths, knees height and hip height were measured for each participant. Since this experiment’s focus was to record human movement disruptions, the XSens suit was used instead of RGB-D cameras to alleviate occlusion problems. The robot joint positions, orientations, forces and torques were streamed as message data in a ROS environment synchronized with the published motion capture suit data. Participants were instructed not to move their feet outside the marked area on the floor in front of them. The two joint frames of the feet were used as fixed reference frames with respect to the robot base.

**FIGURE 3:**
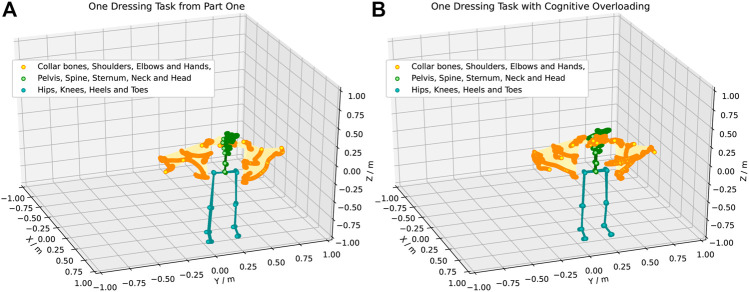
An example of the human movement recorded using the XSens motion capture suit. **(Panel A)** shows the movement of one participant during one dressing iteration performed in Part One. **(Panel B)** shows the movement of the same participant during one dressing iteration performed in Part Two during the *cognitive overloading*.

From the time-series data collected, the data until marker three on Figure 1 were extracted from the rest of the data. This segment of one dressing iteration is represented in the first five images in Figure 1. The position (*x*, *y*, *z*) and the quaternion orientation (*x*, *y*, *z*, *w*) of the right and left arms were used for the human movement projection in a latent space. Figure 3 shows an example of how one of the participants moved during one collaborative dressing task. Sub-Figure 3A shows the human movement recording in Part One. Sub-Figure 3B shows the human movement recording during one dressing iteration with *cognitive overloading*. There is a clear visual difference in the human movement in Sub-Figure 3B when compared to Sub-Figure 3A. The orange and green markers are the features of interest to identify any disruptions in the human movement due to a change in collaborative behavior. The orange markers represent the hands, elbows, and shoulder movement, whereas the green markers represent the collar bones, head, neck, spine, pelvis, and sternum. The upper body movement was considered for the analysis of the type of movements and visualization on a projected latent space. The orange (shoulders, elbows and hands) and green markers (collar bones, head, neck, spine, pelvis and sternum) features were used from the dataset to generate the comparison in the latent space.

A comparison between human and robot movements was implemented to evaluate synchronicity in the collaborative task. Changes in observed parameters during cognitive overloading and distractions can be captured by comparing trajectories during collaboration. The relative entropy Kullback–Leibler (KL) divergence between robot and human arm trajectories is generated from the probability distribution using the sliding window approach. This requires generation of a probability distribution of the right end-effector trajectory *p*(*τ*_*r*_) and the right human arm trajectory representations *p*(*τ*_*h*_). The mean and covariance of each dimension of a trajectory *p*(*τ*) = *p*(*x*_1_, … , *x*_*T*_) are used to create *p*(*x*_*r*_) and *p*(*x*_*h*_) respectively. Hence the KL can be represented as:

$$KL\left(p\left(x_{r}\right),q\left(x_{h}\right)\right)$$

and

$$KL\left(q\left(x_{h}\right),p\left(x_{r}\right)\right)$$

Therefore, the KL divergences is calculated as

$$KL\left(p\left(x_{r}\right.\right)\|q\left(x_{h}\right)=\int p\left(x_{r}\right)\log\frac{p\left(x_{r}\right)}{q\left(x_{h}\right)}dx$$ (1)

### 3.3 User Experience Data Collection

The qualitative user experience data collection is critical for supporting our arguments based on hypothesis derived from human behaviour, action cognition and motor control. We evaluated the collaborative behaviour from the participant’s feedback, particularly how they expressed their experience when their movement was disrupted during the collaborative task. After every set of ten dressing iterations, participants were asked to evaluate their workload during each collaborative task. This qualitative measure was collected using the NASA TLX questionnaire which was scored based on a weighted average of six sub-scales: 1) mental demand, 2) physical demand, 3) temporal demand, 4) performance, (v) effort, and (vi) frustration Hart and Staveland (1988). This measure estimates the impact of *cognitive overloading* and *distractions* and verifies that participants experienced an increase in the mental effort in Part Two and Three compared to Part One of the experiment.

Additional participant feedback was gathered using the PeRDITA (Pertinence of Robot Decisions in joinT Action) questionnaire as presented in Devin et al. (2018). The PeRDITA is inspired by the UX (User eXperience) model presented by Bartneck et al. (2009) in which the interaction is explained in terms of: *“a consequence of a participant’s internal state, the characteristics of the designed system and the context (of the environment) within which the interaction occurs.”* The user’s internal state includes predisposition, expectations, needs, motivation, and mood of the user, while the context of the environment includes social setting, the meaningfulness of the activity, voluntariness of use, and collaboration intention.

The PeRDITA questionnaire assesses several aspects of interaction as shown in Table 1 which form part of the five dimensions of interaction. The *Interaction* dimension quantifies the participants’ behavioral intention, and this dimension is based on the AttrakDiff questionnaire proposed by Lallemand et al. (2015). The *Robot Perception* dimension evaluates how participants perceive the robot and is based on the Godspeed questionnaire as presented in Bartneck et al. (2009). The other dimensions provide insights into how participants perceive joint actions in the robot assistive task and include the *Acting, Verbal* and *Collaboration* dimensions. *Acting* is a measure of the human perception of the decisions taken by the robot. *Collaboration* quantifies the cooperation with the robot in terms of acceptability, usability, and security. No *verbal* communication is used in this experiment. During dressing, people do not tend to use clear verbal communication and instruction can be ambiguous as shown in Chance et al. (2017).

**TABLE 1:** PeRDITA Questionnaire: Questions describing each dimension. Items are evaluated in a scale of 100.

Dimension	Question	Item
Interaction	In your opinion, generally, the interaction was	Negative/Positive
		Complicated/Simple
		Not practical/Practical
		Unpredictable/Predictable
		Ambiguous/Clear
Robot Perception	In your opinion, the robot is rather	Machinelike/Humanlike
		Artificial/Living
		Inert/Animated
		Apathetic/Responsive
		Unpleasant/Pleasant
		Disagreeable/Agreeable
		Stupid/Intelligent
		Incompetent/Competent
Collaboration	In your opinion, the collaboration with the robot to perform the task was	Restrictive/Adaptive
		Useless/Useful
		Unsettling/Satisfactory
		Annoying/Acceptable
		Insecure/Secure
Verbal	In your opinion, robot verbal interventions were	Incomprehensible/Clear
		Insufficient/Sufficient
		Superfluous/Pertinent
Acting	In your opinion, the robot actions were	Inappropriate/Appropriate
		Useless/Useful
		Unpredictable/Predictable

## 4 Results

**FIGURE 4:**
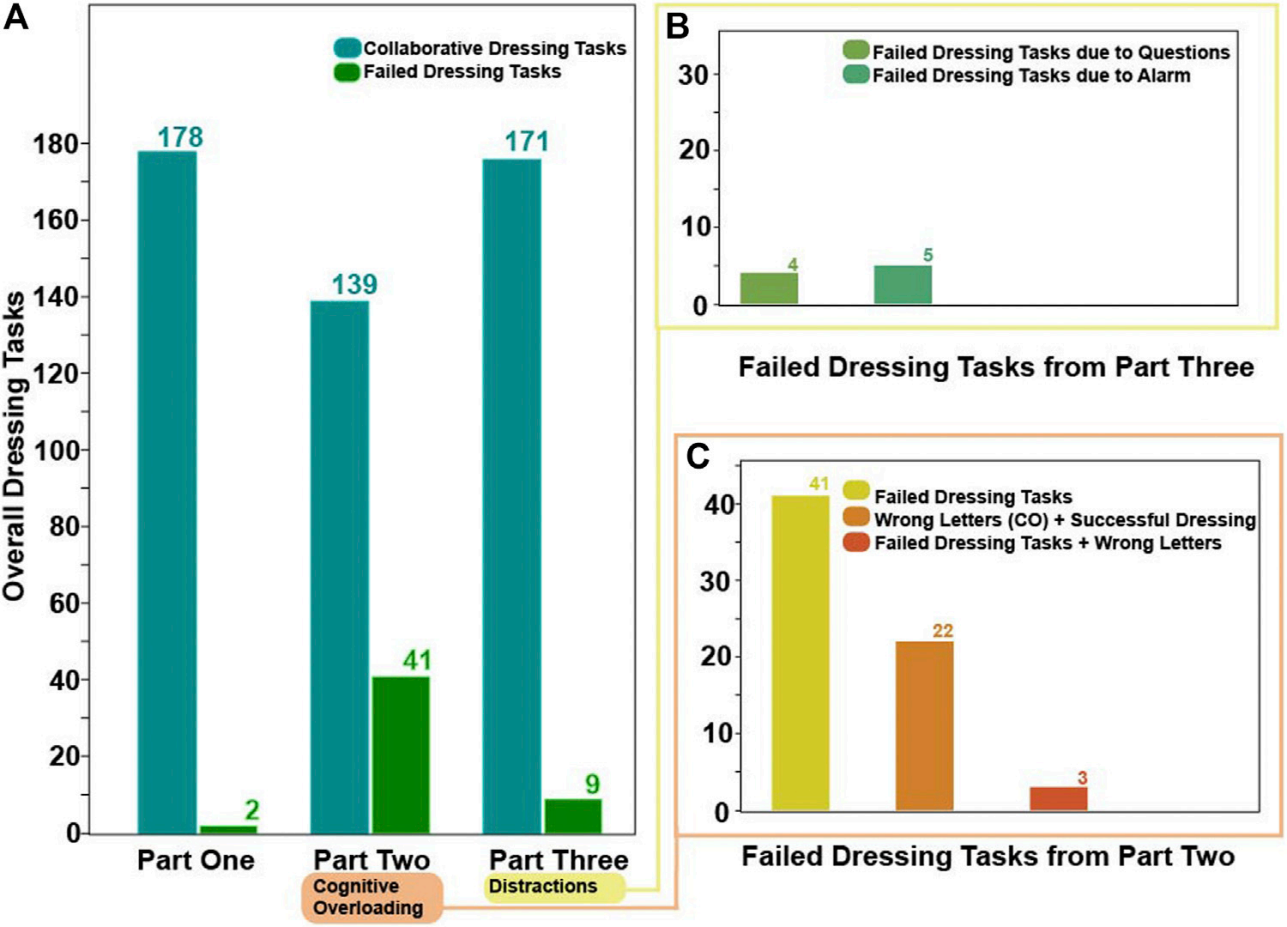
Dressing failure and mistakes count in the different parts of the HRI experiment. **(Panel A)** shows the dressing failure in Part One. **(Panel B)** shows the dressing failures that occurred in Part Three. **(Panel C)** shows the dressing failures and mistakes that occurred during the *cognitive overloading* in Part Two.

Figure 4 shows a breakdown of the dressing failures and mistakes during the 180 dressing iterations for each part of the experiment. In Part One, we recorded two failed dressing iterations. In Part Two, there were 41 failed dressing iterations, whereas in Part Three there were nine dressing failures. In addition to the dressing failures in Part Two some participants failed to memorize the four consecutive letters appearing (and disappearing) from the monitor correctly. This means that a total of 22 mistakes occurred during the rest of the 139 collaborative dressing iterations in Part Two. Three out of the 41 dressing iterations were both mistakes in recalling the letters as well as dressing failures. From the failures that occurred in Part three, 5 were attributed to the fire alarm and 4 to the random questioning. The term dressing failure means that participants missed the opportunity to synchronize their movement with that of the robot to enable the insertion of the right arm or left arm in the jacket. This suggests that the *cognitive overloading* might have hindered the participant’s ability to adapt and collaborate with the robot.

### 4.1 Evaluation of User Experience and Work Load

**FIGURE 5:**
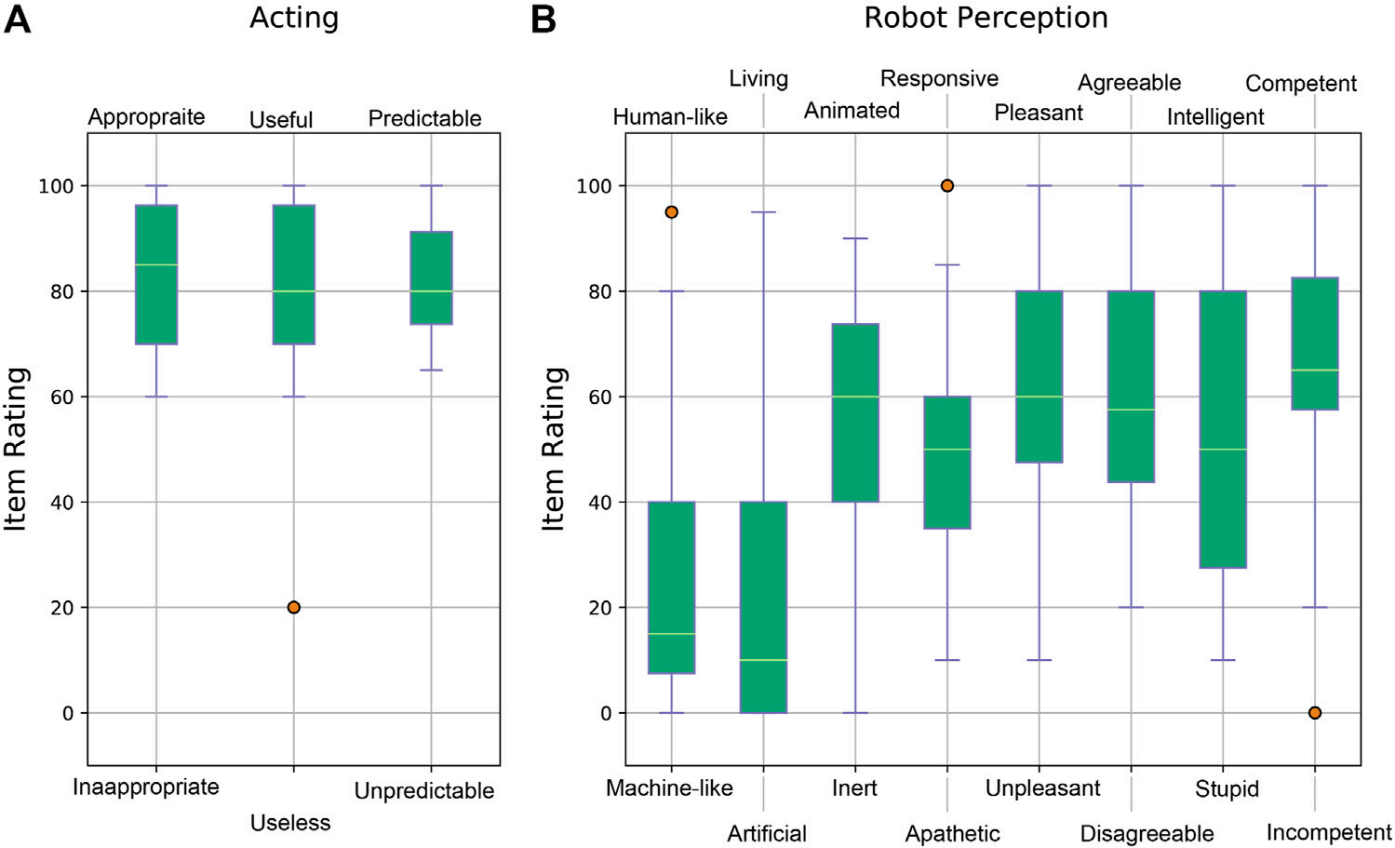
Results of the PeRDITA questionnaire for the *Acting* and *Robot Perception* dimensions. **(Panel A)** shows the item ratings forming part of the *Acting* dimension, and **(Panel B)** shows the item ratings for the *Robot Perception* dimension.

**FIGURE 6:**
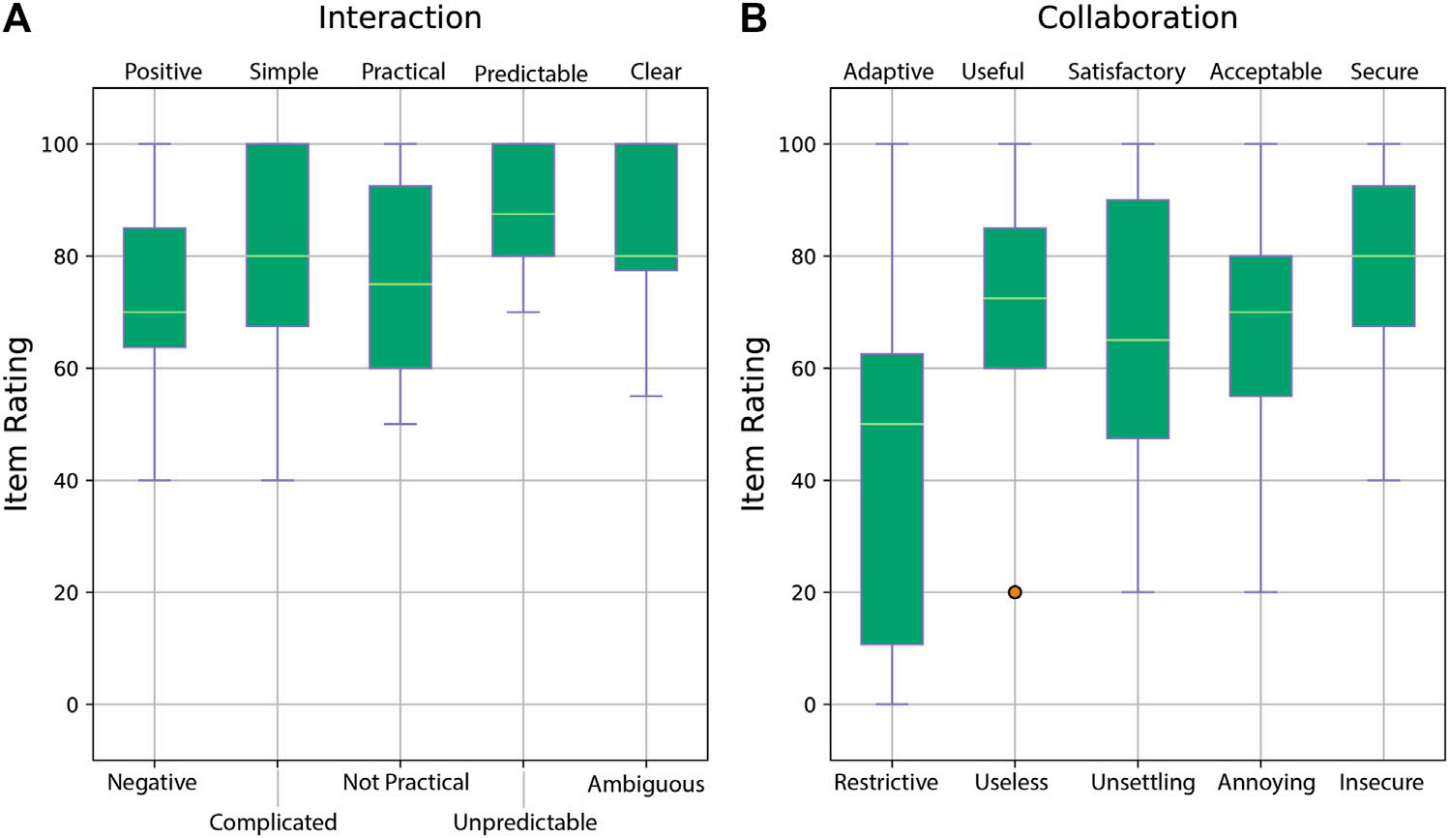
Results of the PeRDITA questionnaire for the *Interaction* and *Collaboration* dimensions. **(Panel A)** shows the item ratings forming part of the *Interaction* dimension, and **(Panel B)** shows the item ratings for the *Collaboration* dimension.

As described in Section 3, the qualitative data collection aims to understand the participants’ experiences of the disruptions in collaboration during the dressing task. Participants were asked to answer the PeRDITA questionnaire, explained in Table 1 by marking from 0 to 100 each item in the dimensions of the interaction. The PeRDITA questionnaire aims in getting feedback about how participants perceive their collaboration with the robot during the dressing task. The PeRDITA results obtained from our controlled HRI experiment is shown in Figure 5 and Figure 6.

Sub-Figure 5A shows the box-plots for the dimension of the interaction of *Acting*. Participants were asked to rate the interaction in terms of appropriateness, usefulness and predictability. Overall, the participants describe the collaborative task as *useful, predictable* and *appropriate*. The score of the item *Predictable* in the *Acting* Dimension of Interaction suggests that participants perceived the robot’s trajectory to be predictable in the context of the collaborative task. Such a score was recorded even though the participants failed to maintain a collaboration behavior during unexpected events. Sub-Figure 5B shows the box-plots for the *Robot Perception* dimension. The Baxter Research robot was described as *machine-like* instead of *human-like* and *artificial* instead of *living*. An average score between 50 and 60 was given to the items of *animated, responsive* and *pleasant*. The majority of the 18 participants described the robot as *agreeable* and *competent*. On the other hand, the item of *intelligence* during the collaborative task had the largest variance from all items. A few participants did perceive the robot as *human-like*, *responsive* and *not competent*. The robot was classified as appropriate to carry out the collaborative task even though the robot did not adapt to the participants’ changing behavior.

Figure 6 shows results from the rating of the *Interaction* and *Collaboration* dimensions of interaction. Overall, the *Interaction* dimension results shown in sub-Figure 6A is described as *positive, simple, practical, predictable* and *clear*. Although the verbal dimension was non-existent in the experiment, the interaction dimension achieved a high score. This suggests that verbal interaction was not considered as important in being able to achieve this physically assistive interaction. The trajectory executed by the robot was implemented in such a way as to mimic humans helping each other to get dressed. The participant’s ratings of the *Collaboration* dimension are shown in Sub-Figure 6B. The collaboration dimensions in the controlled HRI experiment were highly regarded as *secure, acceptable,* and *useful*. A lower average and higher variance are recorded in the *Satisfactory/Unsettling* item. This significant variance might be due to the mistakes and dressing failures during the collaborative task. An even higher variance and a lower average are seen in the *Adaptive/Restrictive* item. These low ratings can be attributed to the lack of adaptation from the robot side. The uncertainty in participants’ ratings could be attributed to the fact that they might think they have failed in collaboration due to unexpected events. The highest rating in the *Adaptive* item is from one of the participants who did not have any dressing failures in Part Two. The participants who did not let the *cognitive overloading* affect their collaborative behavior might have gotten the impression that the overall collaboration was more adaptive. As such, these participants might perceive the robot as more *adaptive* when compared to the other participants’ experience. The lowest rating of the *Adaptive* item is given by the participants who failed both the dressing task and gave the wrong answers to the *cognitive overloading* task. Hence, the variance in rating the *Adaptive* item in the PeRDITA questionnaire could be linked to the varied effect of the *cognitive overloading* on different participants. It is essential to note that the PeRDITA questionnaire was evaluated after Part One, Two and Three were finished. We can only argue that these results are an overall evaluation that includes the cases with no cognitive overloading and distractions. Any observation of these items can indirectly be an effect of the cognitive overloading or distraction because these were still part of the overall interaction. Still, a direct conclusion cannot be made with respect to the individual parts of the experiment.

**FIGURE 7:**
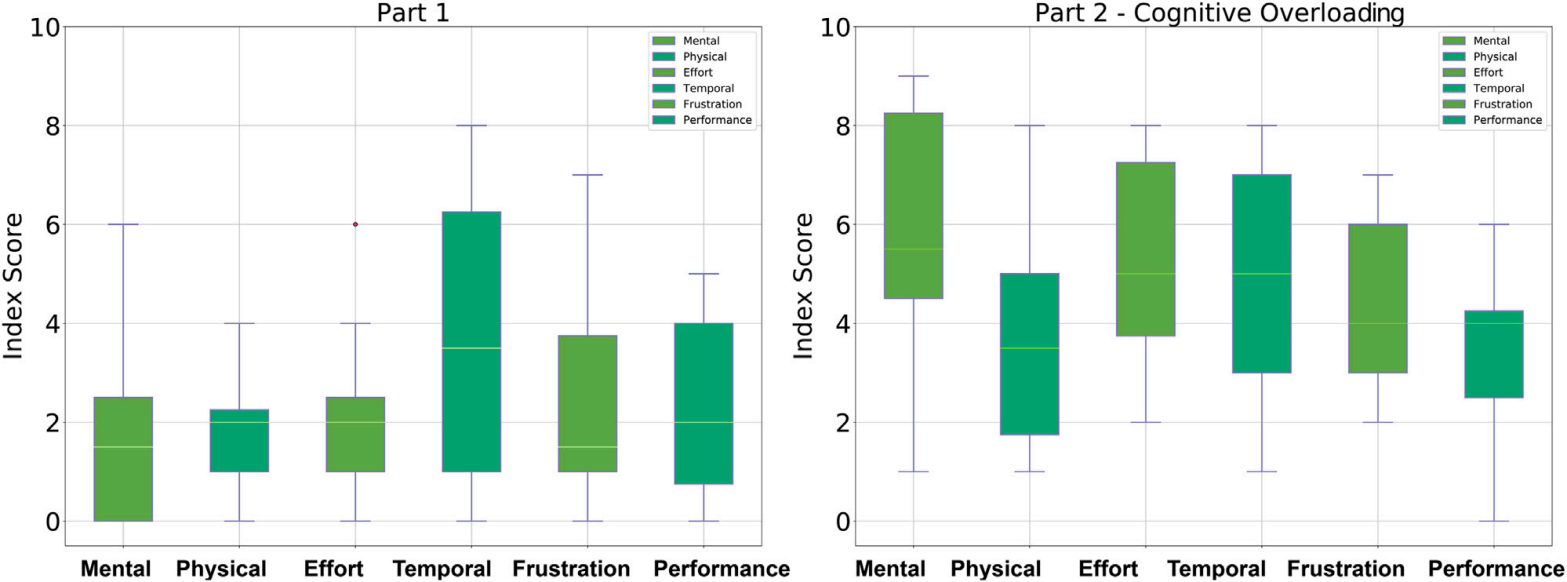
Box-plots showing the NASA TLX data collection from participants. **(Panel A)** shows the workload in Part One compared to **(Panel B)** which represents the increased workload due to *cognitive distractions*.

**FIGURE 8:**
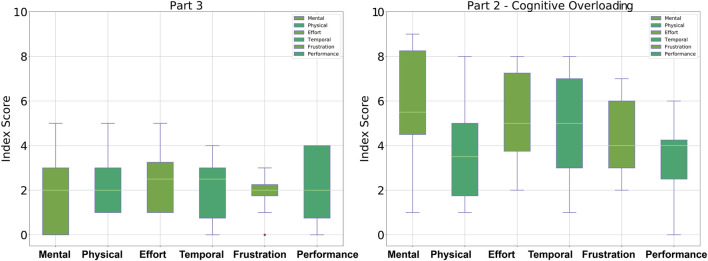
Box-plots showing the NASA TLX data collection from participants. **(Panel A)** shows the workload in Part Three compared to **(Panel B)** which shows the workload from participants during from Part Two.

In order to evaluate the participant’s perceived mental effort during the different parts of the controlled HRI experiment, the NASA TLX was used. After ten dressing tasks, meaning after each part of the experiment, the participants were asked to evaluate their mental, physical, temporal demand, effort, frustration, and performance in collaborating with the robot for the assistive dressing task. Figure 7 shows the results obtained from participants after performing ten dressing iterations in Part One of the experiment compared to the task load demanded during Part Two of the experiment. Figure 8 shows the participants perceived load during Part Three compared to load perceived during Part Two of the controlled HRI experiment.

Overall, participants described Part Two of the controlled HRI experiment as the highest in terms of workload, particularly in mental, temporal demand and effort in executing the collaborative task. Initially, participants struggled to balance their attention between collaborating to perform the physically assistive task and the *cognitive overloading*. This cognitive overload caused the participants to focus less on when and how to move to maintain a collaborative behavior with the robot. The occurrences of the failures and mistakes in Part Two suggest that when participants could not manage the intrinsic cognitive load, they seem to prioritize either the collaborative tasks or memorize the letters on the monitor. The controlled HRI experiment in Part Two required participants to balance their attention between the temporal requirements of the unexpected events and the collaborative task. The only two participants who managed to carry out Part Two of the experiment without dressing failures gave the most wrong answers in comparison with all the other participants, indicating that their priority was on the dressing task. Furthermore, the highest measure of frustration is observed among the participants with the highest combined dressing failures and wrong answers count. The participants who both made errors in the dressing task and gave a wrong answer at different instances were also the ones who rated the temporal demand the highest. In Part Three, the distractions reduced control over the intrinsic cognitive load and caused participants to deviate from the plan of performing the assistive task.

The overall increase in the workload described by participants from the NASA TLX supports our hypotheses for our design of the controlled HRI experiment. The results suggest that controlling the occurrence of the *cognitive overloading* and *distractions* during the collaborative task managed to trigger the unbalancing in the intrinsic, extraneous and germane loads. The general overview of these results shows that no matter how familiar the participants were with the task, the *cognitive overloading* and *distractions* in Part Two and Part Three caused disruption to the participants’ movements. Therefore, this controlled experiment shows that in spite of an already known interaction, unexpected events may lead to variations in the performance of experienced users interacting with a robot. Such disruptions in human movement will be critical in collaborations that require synchronicity. Consequently, this highlights the importance of analysing and evaluating the changes in human collaborative behaviors and human movement, particularly during assistive tasks.

### 4.2 Evaluation of Collaborative Human Movement Disruptions

Through the controlled HRI experiments we were able to evaluate the effect of action cognition on motor control to assess how the change in the human collaborative state disrupts the human movement under *cognitive overloading* and *distractions*. As shown in Figure 3, there is a clear difference in disrupted human movement between the different parts of the experiment. From each part of the experiment, the movement of both arm poses was extracted from the recorded data. The data points are the joints marked as collar bones, shoulders, elbows and hands in Figure 3. Overall, 753,910 arm poses have been recorded from a total of 23 joints from the entire human posture. Each joint comprises seven features (position and orientation) as described in Section 3.2.

**FIGURE 9:**
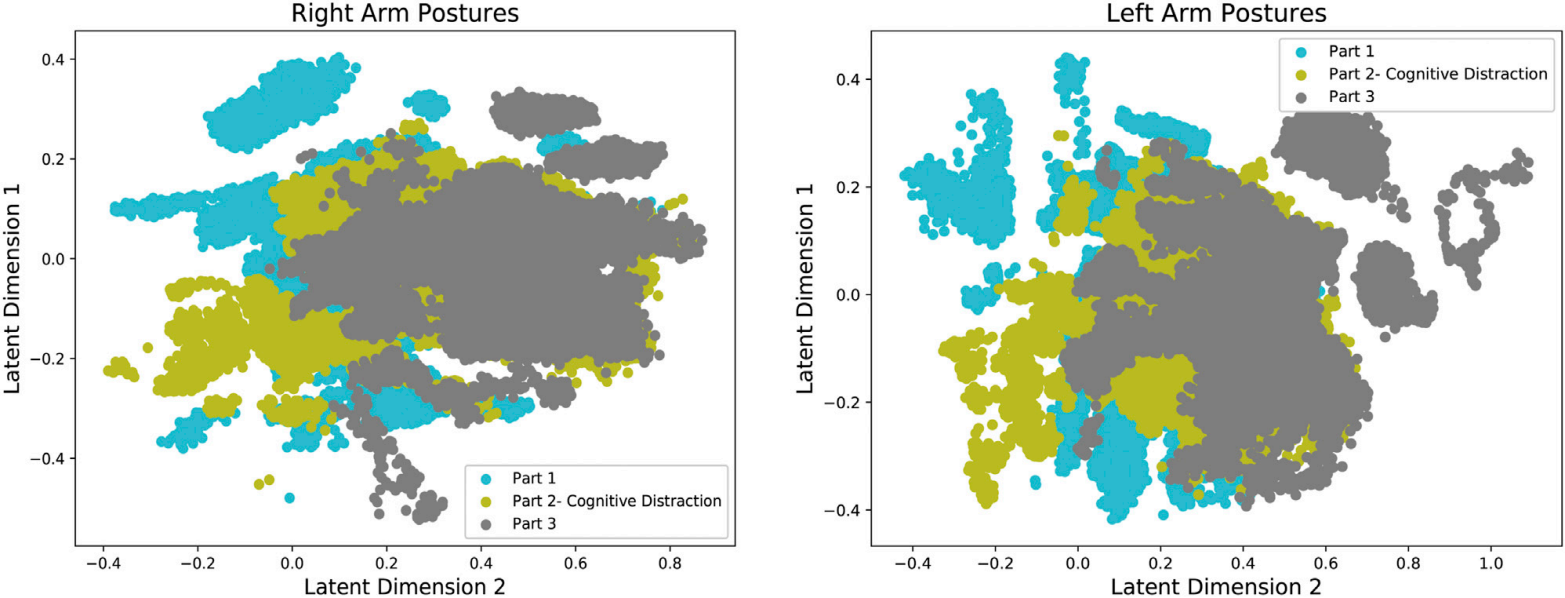
The 2-D latent space representation of the 9-D right arm posture data for all participants, produced with GP-LVM. The different colored projections denote arm movements during cognitive overload.

**FIGURE 10:**
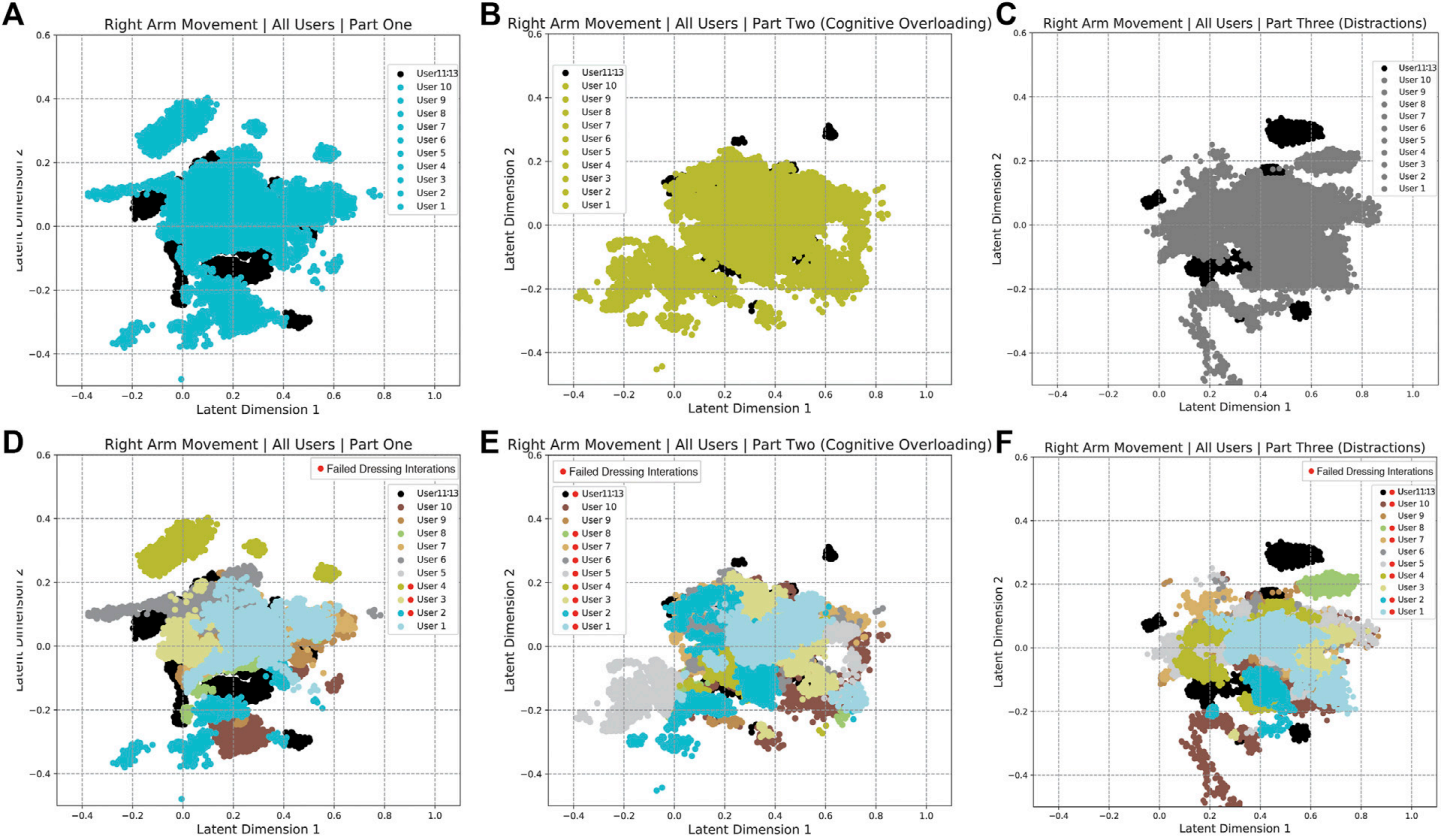
Separated Latent Space representation of the right arm movements for Part One **(A,D)**, Part Two **(B,E)** and Part Three **(C,F)**. **(Panels A–C)** show the 2-D latent space representation for all the participants. **(Panels D–F)** shows the representation of ten individual participants. The participants marked with a red dot in the legend had dressing failures in their dressing iterations.

**FIGURE 11:**
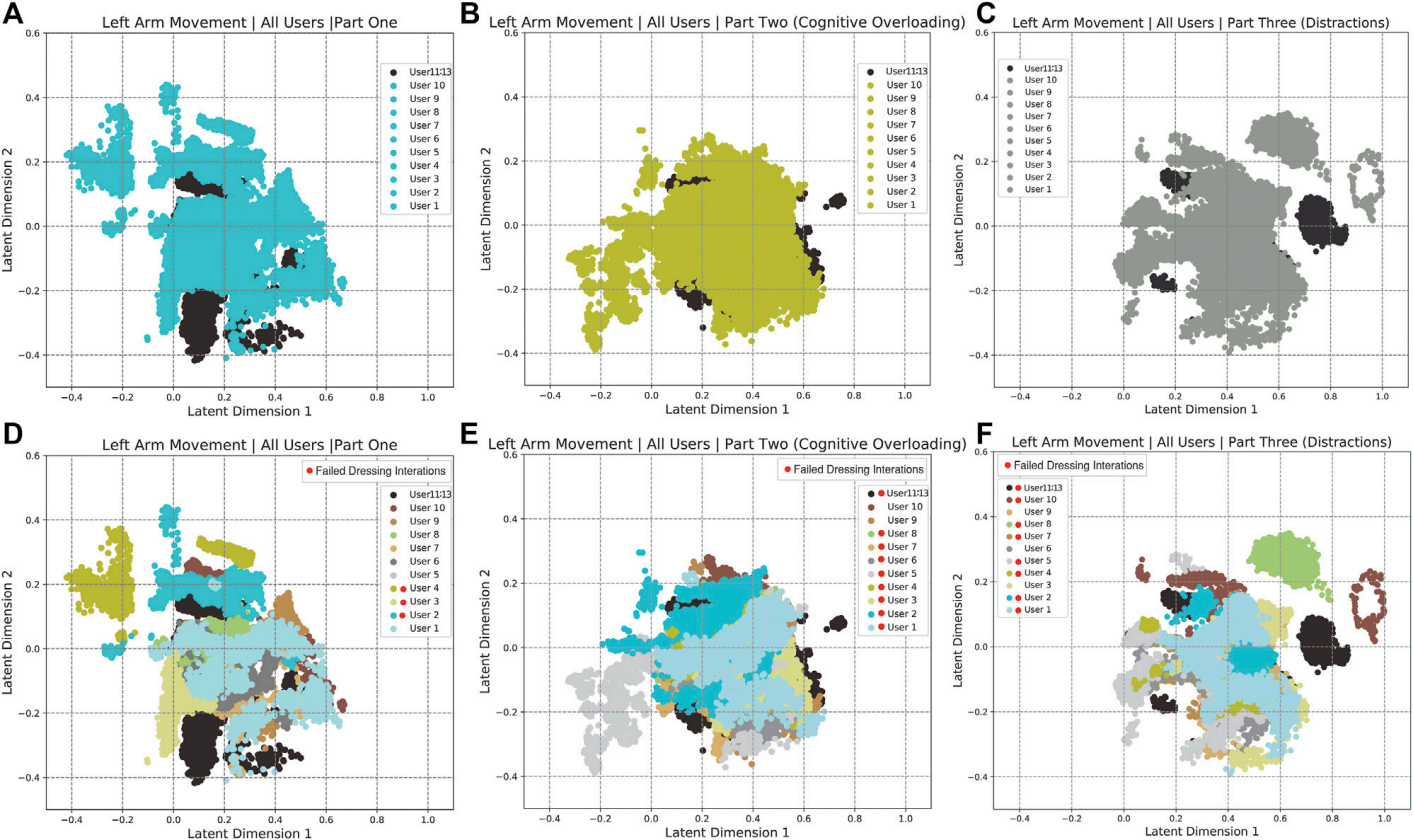
Separated Latent Space representation of the right arm movements for Part One **(A,D)**, Part Two **(B,E)** and Part Three **(C,F)**. **(Panels A–C)** show the 2-D latent space representation for all the participants. **(Panels D–F)** shows the representation of ten individual participants. The participants marked with a red dot in the legend had dressing failures in their dressing iterations.

**FIGURE 12:**
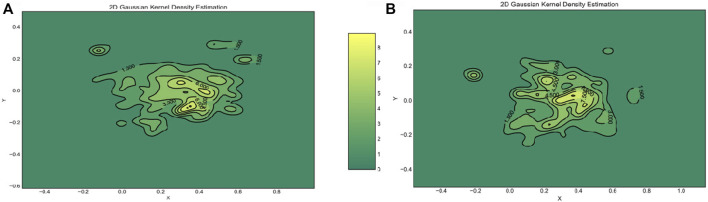
Plot of the probability distribution function using a Gaussian 2D KDE. **(Panel A)** shows the density surface for the right arm of the participants. **(Panel B)** shows the density surface for the left arm of the participants. Figure changed to 2D plot with color gradient instead of 3D.

The human arm movement data were projected into a 2-D latent space using the GP-LVM Gao et al. (2015). The 2-D latent space reveals differences in human movements between different parts of the experiment. Figure 9 shows a map of the right and left arm poses in 2D space with three sub-spaces in different colors, indicating three different parts of the experiment. Figures 10, 11 show right and left arm latent space, respectively, for each part of the experiment. From Part Two in Figures 10, 11, the variation in the right arm movement is greater than the variation in the left arm movement. During this part of the experiment, the *cognitive overloading* was overwhelming the participants because they could not process the information presented to them while also simultaneously participating in the assistive task. It was observed that during the initial part of the dressing task (until marker two in Figure 1), participants got agitated by quickly trying to move the right arm first but failing to synchronize their movement as they did in Part One. It was observed that participants who failed to insert the right arm in the jacket gave up trying to insert the left arm in the jacket, hence the fewer variations in the latent space. The overall representation of the projection indicates that participants used similar movements, somewhat restricted to a particular subspace shown in the central part of the graphs. This subspace can be assumed to show fundamental arm poses during collaborative behavior. Figure 12 shows density distribution of the projected latent space from Figure 9. The lighter colors represent higher densities of human movement during the whole experiment. Figure 12 shows how the majority of the movement is centered on the latent space, meaning that most of the movement during the assistive task was consistent. Figure 4 shows that overall the success count of collaborative tasks was higher than the failed dressing task. This higher success rate suggests that a higher density can be attributed to a collaborative region rather than the non-collaborative states. Hence the lower density range areas on Figure 12 should be the regions where a significant difference between Part One, Two and Three should be observed, as seen in Figure 10.

**FIGURE 13:**
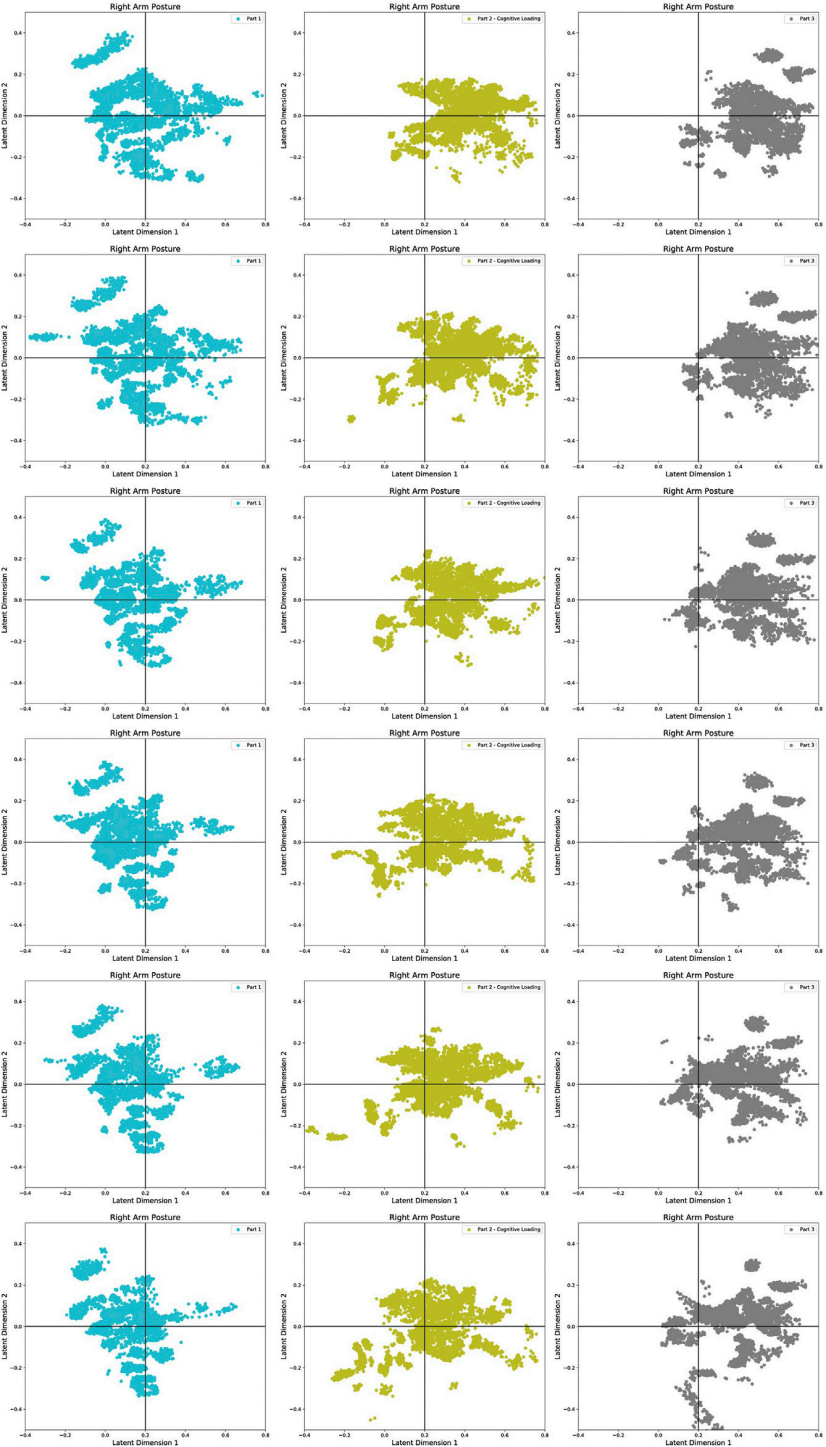
Comparison of latent space projections during different parts of the experiments along the progression in the dressing sequence. The progression of Part One is represented by the LHS column, Part Two by middle columns and Part Three by the RHS column.

A timeline of latent space changes is shown in Figure 13, demonstrating variations in the right arm movement during all three parts of the experiment. The latent space is divided into quadrants for ease of analysis and comparison of the different parts of the experiment. The first column shows the latent space projection of the Part One of the experiment. There is a broader distribution at the first iterations of the controlled HRI experiment. At this stage, participants were starting to learn how to collaborate and build a plan for the collaborative task with the robot. In the second and third columns, the distribution of the projected points is less spread because over time, presumably as the participants were able to learn the task and so undertake it in a more controlled and automated learned manner. However, this automated or learned motion is disrupted by *cognitive overloading* in Part Two depicted by projections in column two. The main difference from the projected movement in column one is the top right corner. The projected movement in this quadrant is associated with the timestamp when dressing failures occurred during the collaborative task. In column three, the same can be seen in all the top right quadrants. Additionally, there is a variation in the bottom row of column three compared to columns two and one. These outliers in the latent space in Part Three are related to the nature of the unexpected events. For example, the fire alarm sound caused some participants to move away from the collaborative task; the random questioning provoked some participants to stop collaborating and move back to the starting position. These type of movements are relatively different from the ones shown in columns one and two.

### 4.3 Synchronicity Between the Human and Robot Arm Movement During the Collaborative Task

**FIGURE 14:**
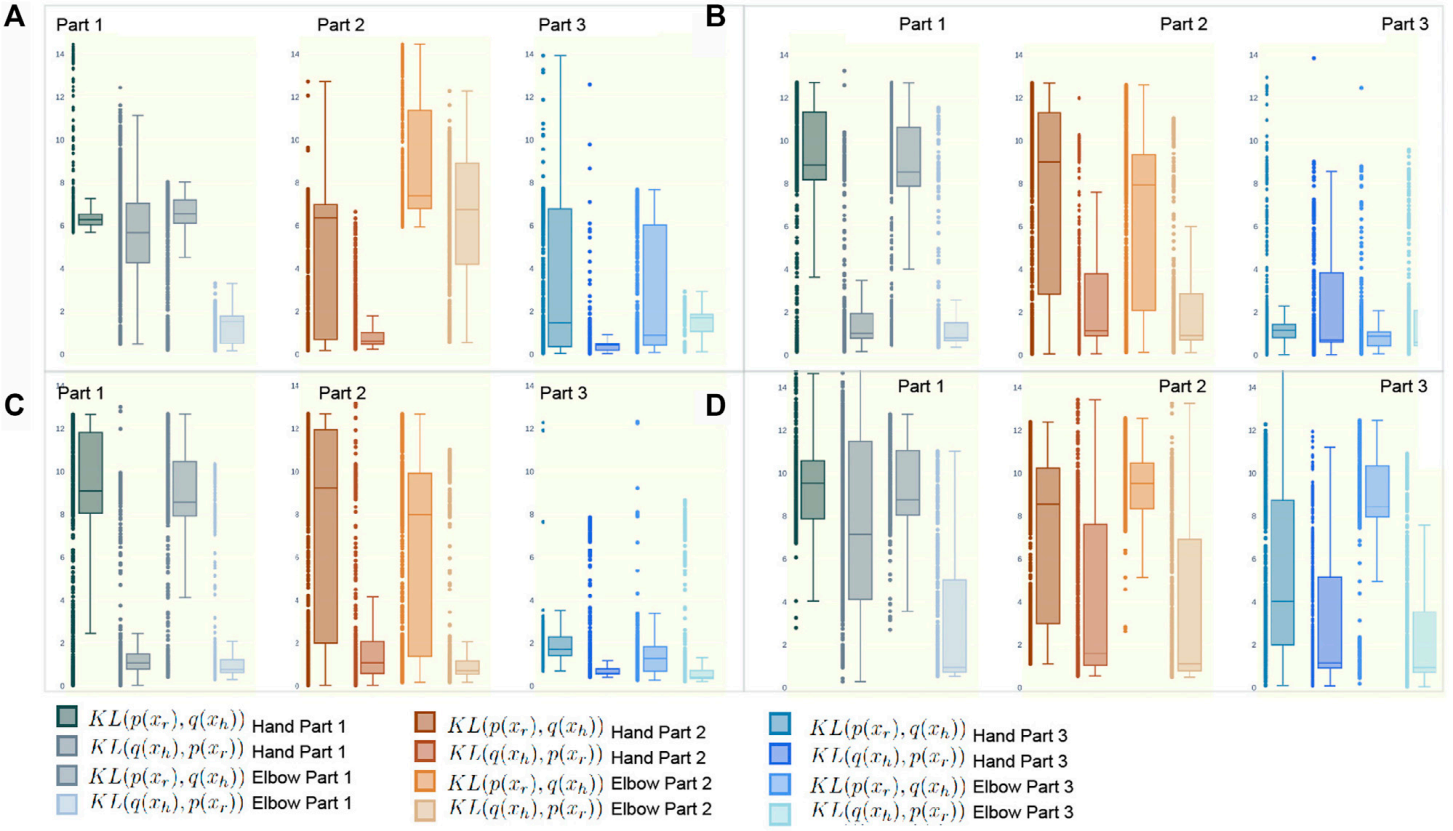
KL divergence is represented as a measure of deviation between the robot end-effector and the hand (first two box plots in each subplot) and elbow of four participants panel **(A–D)** in the assistive task. For each participant there is a cooperation measure computed for each part of the experiment. The brown box plots shows a measure of the cooperation during cognitive overloading.

Figure 14 shows the relative entropy between the robot trajectory and the human arm. The *KL*(*p*(*x*_*r*_), *q*(*x*_*h*_)) and *KL*(*q*(*x*_*h*_), *p*(*x*_*r*_)) were calculated for two joints of the right arm of four participants with respect to the robot’s right end-effector. Each subplot in Figure 14 is a measure of relative synchronicity between the robot and the human arm. The four sub-figures (A, B, C and D), show a lower measure for a more synchronous movement between the robot and the participants. In sub-Figure 14A, the biggest lack of synchronicity is found in the middle box-plots associated with Part Two of the experiment. These low synchronicity measures (highest divergence) can be seen in all the middle box-plots of each sub-figure in 13. The box-plots of Part Three (the third column in sub-Figure 14A) shows an average of a more synchronous movement when compared to the first column showing movement from Part One. This suggests that participants over time are improving their ability to have an automated plan of performing the task as mentioned in Section 2.2 and Section 3.1. The high variance in the third column of sub-Figure 14A is due to the *distractions* in Part Three. Similarly, this can also be seen in sub-Figure 14B and sub-Figure 14D. On the other hand, the third column in sub-Figure 14C, shows the most synchronous movement out of all the 12 sets of plots. This is because the participant performing the collaborative task did not have any dressing failures in Part Three of the experiment whilst already having the experience of performing more than 20 dressing iterations in Part One and Part Two.

## 5 Discussion

The main contribution of this study is the analysis and quantification of disrupted human movements during a physical HRI task. The effects of the disruptions were further confirmed through the qualitative evaluation of the user experience. In literature related to close-proximity robot-assistive tasks, consistent human movement during physical collaboration is always assumed. This assumption of a continuous commitment to the collaborative task from the human side can pose a safety risk in a real dynamic environment. The research presented in this paper is an important step towards recognizing and characterizing the breakdown in collaborations that can occur during cognitive overloading and distractions while performing assistive tasks.

The timeline (see Figure 1) and temporal layout (see Figure Figure2) of the HRI experiment devised for this study are based on the literature on human behavior, action cognition and motor control, and therefore will be useful for other researchers conducting similar studies. Results collected through the NASA TLX and PeRDITA questionnaire further help validate the HRI experiment’s methodology. The dressing failures, mistakes (see Figure 4), and the qualitative feedback from the participants were found to correspond to the quantitative human motion data. Parts Two and Three of the experiment were specifically designed to disrupt the way participants initially learned to perform the collaborative task. The results shown in Figures 7, 8 suggest that the *cognitive overloading* in Part Two can lead to unmanageable intrinsic cognitive load and large extrinsic cognitive loads. In Part Three of the experiment, the recorded data shows slightly less movement than in Part Two which demonstrates that participants managed the new information in the environment slightly better than the first time (in Part Two). The participants learned how to collaborate in Part One, but the unexpected events continuously challenged the germane load during the experiment. The NASA TLX (see Figure 7) for Part One shows a higher temporal demand than Part Three. The difference is most likely associated with the fact that during these initial ten iterations of the experiment participants were still trying to understand the dynamics of the collaboration, and build their own approach to performing the task. The breakdown of collaborative behavior is also represented in the PeRDITA results. Although participants overall described the Interaction as *simple, predictable* and *clear*, there was uncertainty in describing the Collaboration as *adaptive*. The participants collaborating with the robot, particularly in the Part Two, had the impression that they failed to adapt—they learned to carry out the task in Part One but failed to maintain synchronicity during *cognitive overloading*. All participants either made a mistake or a dressing failure during the *cognitive overloading*. The participants who did not fail in the dressing task answered most questions incorrectly during the *cognitive overloading*. The results confirm that even when familiar with the task, participants can lose concentration when unexpected events occur, resulting in a loss of interaction synchronicity.

The failures and mistakes in Part Two and Three were caused by the change in the human collaborative state. The projection of the human movement on the latent space shows the learning process over all three parts of the experiment. Despite almost no dressing failures in Part One, there is a more significant dispersion of points in the latent space because participants were still trying to learn how to execute the task. The germane cognitive load is described as an effort to construct a mental model of the task. In Part One participants were building a mental model of the, so this load was likely to be high. This learning process is reflected in the high variance of the temporal effort from the NASA TLX (see Figure 7). The high temporal effort indicates that participants were learning how to synchronize their movements with the robot. The projections for Part Two and Part Three show that disrupted movements moved away from the center of the 2D latent space, which was not the case with non-disrupted movements. The projections show that when the cognitive loads are unbalanced (as in Part Two and Three), the ability to retrieve the knowledge of how to perform the task is affected, impacting human motor control. The projected 2-D latent space has movements performed when learning to collaborate, movements performed in synchronicity with the robot, and movements disrupted due to unexpected events. This change in synchronicity between the robot and the human is also captured using the KL relative entropy shown in Figure 13. Some form of synchronicity measure could be calculated to separate typical movements from movements performed while learning to collaborate or disrupted due to unexpected events. If synchronicity can be reliably identified and characterized then we could be confident in applying a collaborative robot mode only when the human is in a collaborative state. Additionally, the collaborative state of each participant requires some form of personalization. By looking at the user experience and mistakes as presented in Section 4, it is evident that every participant can react entirely differently to external disturbances. The impact of cognitive overloading and distraction on human motor control is distinctive due to its complex form. Therefore the collaborative and non-collaborative state of the assistive robot would still require some form of personalization to cater for these differences based on the specific end-user.

Our future work will focus on creating a synchronicity measure that quantifies the level of collaboration between the human and robot in latent space projections. This will be followed up by using this measure to adapt close-proximity interactions while ensuring that collaboration is taking place. Therefore, a synchronicity measure could ensure that robot adaptation can be achieved safely and realistically. In general, researchers should ensure that similar studies are run with cognitively and physically impaired individuals and people with dementia since these are important potential beneficiaries of such assistive technologies. The potential risks identified in this study requires an experimental methodology that provides realism, experimental rigour and high fidelity of the human-robot collaboration in real context environments and scenarios. Only in this way can we start evaluating how safely and realistically we can deploy such assistive technologies.

## Data Availability

The datasets presented in this article are not readily available because Ethics Committee forbids that this data is used by anyone other the Author. Requests to access the datasets should be directed to antonella.camilleri@uwe.ac.uk.
